# Effects of Metformin on Clinical Outcomes After Acute Ischemic Stroke in Patients with Type 2 Diabetes Mellitus: A Systematic Review and Meta-Analysis

**DOI:** 10.3390/jcm15197532

**Published:** 2026-09-28

**Authors:** Ting-Yen Chen, Yu-Chi Su, Yu-Ching Lin

**Affiliations:** 1 Department of Physical Medicine and Rehabilitation, National Cheng Kung University Hospital, College of Medicine, National Cheng Kung University, Tainan 70428, Taiwan; 2 Department of Physical Medicine and Rehabilitation, College of Medicine, National Cheng Kung University, Tainan 70428, Taiwan

**Keywords:** ischemic stroke, metformin, modified Rankin Scale

## Abstract

**Objective**: This study investigated the effects of metformin on clinical outcomes after acute ischemic stroke in patients with type 2 diabetes mellitus (T2DM). **Methods**: PubMed, Embase, Web of Science, and the Cochrane Library were systematically searched for relevant English articles published from database inception to 6 April 2026. Moreover, the reference lists of eligible articles were searched. Studies were included if they focused on patients with T2DM and acute ischemic stroke, indicated whether the patients used metformin before stroke onset, and reported the patients’ modified Rankin Scale (mRS) scores after stroke onset. Studies lacking adequate outcome data were excluded. Two reviewers independently extracted relevant data—for example, study details, clinicodemographic characteristics, and poststroke outcomes (mRS scores, National Institutes of Health Stroke Scale [NIHSS] scores, symptomatic intracranial hemorrhage [sICH], and mortality). A senior reviewer was consulted in cases of between-reviewer disagreements. The primary outcome was poststroke mRS score (functional outcome), and the secondary outcomes were poststroke NIHSS scores (neurological severity), sICH, and mortality. **Results**: Eleven studies met the inclusion criteria. Most studies reported better functional outcomes (mRS scores) and lower NIHSS scores for metformin users than for nonusers. Some studies indicated reduced rates of sICH and mortality among metformin users than among nonusers. Sensitivity analyses generally supported the benefits of metformin. Caution should be exercised when interpreting these findings because the results might have been influenced by variations in study designs, stroke classifications, and renal function reporting. **Conclusions**: In patients with T2DM, metformin may reduce stroke severity and improve poststroke functional outcomes. To the best of our knowledge, this meta-analysis is the first to investigate the association between metformin use and neurological severity, estimate adjusted ORs for favorable functional outcomes, and highlight publication bias on this topic.

## 1. Introduction

Type 2 diabetes mellitus (T2DM) is a chronic metabolic disease with a high global prevalence [1]. The prevention and treatment of T2DM are crucial because the disease is associated with numerous complications, such as coronary artery disease [2], chronic kidney disease (CKD) [3], and stroke [4,5]. Stroke is a leading cause of disability and mortality worldwide [6]. Among the patients with stroke, those with T2DM often experience severe neurologic problems and poor functional outcomes [7,8].

Metformin, an oral antidiabetic drug (OAD) with a low risk of hypoglycemia, is the first-line treatment for patients with T2DM without severe CKD [9]. In addition to exerting antidiabetic effects, metformin reduces the risks of coronary artery disease [10], CKD [11], neurodegenerative diseases [12], and stroke [13].

In the last decade, metformin has garnered considerable attention from ischemic stroke researchers because it has yielded promising results in animal models [14,15]. In mouse models of cardiac ischemia/reperfusion, metformin attenuated apoptosis, mitochondrial dysfunction, and microglial hyperactivation in the brain [14,15]. In a mouse model of neonatal stroke, the drug promoted motor and cognitive recovery by activating neural precursor cells [16]. Its neuroprotective effects are likely attributable to its activation of adenosine monophosphate-activated protein kinase, which reduces oxidative stress and promotes angiogenesis and neurogenesis [17,18,19].

Evidence indicates that metformin can improve ischemic stroke outcomes. Three meta-analyses have reported that metformin improves functional outcomes and reduces the risks of mortality and symptomatic intracranial hemorrhage (sICH) after ischemic stroke [20,21,22]. However, several key questions remain unanswered in this emerging field. First, does metformin reduce neurological severity at stroke onset? Because of its apoptosis-attenuating effects, metformin is likely to reduce neurological severity at stroke onset. Second, in clinical practice, most patients with T2DM use multiple OADs. Does the neuroprotective effect observed in such patients stem from metformin specifically or from other OADs? Finally, does discontinuing metformin after stroke diminish its neuroprotective effects? Considering its proposed role in functional recovery, continued metformin use may influence functional outcomes in stroke survivors.

In this systematic review and meta-analysis, we investigated the association between metformin use and ischemic stroke outcomes, namely, functional outcomes, neurological severity, sICH, and mortality. In addition, we explored potential moderators of this association—for example, concomitant use of other medications, dosage of metformin, and poststroke continuation of metformin.

## 2. Methods

### 2.1. Guidelines and Registration

This systematic review adhered to the Preferred Reporting Items for Systematic Review and Meta-Analysis guidelines (Table S1) [23]. The study protocol was registered with the International Platform of Registered Systematic Review and Meta-Analysis Protocols (registration number: INPLASY2024110108).

### 2.2. Eligibility Criteria

We included studies involving patients with T2DM and acute ischemic stroke that indicated whether the patients used metformin before stroke onset and reported the patients’ modified Rankin Scale (mRS) scores after stroke onset. We excluded studies that lacked adequate outcome data—that is, articles not reporting the mean ± SD, median (IQR), or (95% CI) values.

### 2.3. Search Strategy

PubMed, Embase, Web of Science, and the Cochrane Library were systematically searched for relevant English articles published from database inception to 6 April 2026. Moreover, the references of selected articles were manually searched to identify additional eligible studies. The key search terms were “metformin” AND “stroke” AND “modified Rankin Scale”. Table 1 presents the full search strategy.

### 2.4. Study Selection and Data Extraction

Two reviewers (TYC and YCS) independently screened the titles and abstracts of relevant studies. If a consensus could not be reached through discussion, a senior reviewer (YCL) was consulted to make the final decision. The following data were extracted from selected studies by using a standardized data collection sheet: the first author’s name; publication year; study design; and participants’ demographic characteristics (age and sex), clinical characteristics (reperfusion therapies, serum glucose levels, serum HbA1c levels, and renal function), and poststroke outcomes (National Institutes of Health Stroke Scale [NIHSS] scores, mRS scores, intracranial hemorrhage prevalence, and mortality). When necessary, the authors of relevant articles were contacted to clarify uncertainties. For studies reporting median (IQR) values instead of mean ± SD values, we adopted the quantile estimation approach proposed by McGrath et al. [24].

### 2.5. Quality Assessment

The quality of the included studies was assessed using the Joanna Briggs Institute Critical Appraisal Checklist for Cohort Studies [25]. Between-reviewer disagreements were resolved through discussion. The senior reviewer (YCL) made the final decision if a consensus was not reached. RevMan (version 5.3; Cochrane, London, UK) was used to visualize the risk of bias through a graph and summary table.

### 2.6. Study Outcomes

The primary outcome was poststroke functional outcome, assessed using the mRS. The secondary outcomes were neurological severity, assessed using the NIHSS; sICH after intravenous thrombolysis (IVT); and mortality.

### 2.7. Statistical Analysis

All studies reporting the outcomes of interest were included in the quantitative synthesis. Effect sizes were pooled using a random-effects model. Categorical data are presented as ORs with 95% CIs, whereas continuous data are presented as standardized mean differences (SMDs) with 95% CIs. For articles missing mean ± SD values, we used available *p* values and sample size information [26]. Heterogeneity across the included studies was assessed using the *I*^2^ statistic, with values of >50% indicating significant heterogeneity [27]. Publication bias was evaluated using funnel plots and the Egger test [28]. A 2-tailed *p* value of <0.1 was considered significant [29].

A subgroup analysis was performed to determine whether the mRS score at 3 months after stroke onset (3-month mRS score) differed between single-center and multicenter studies. Nonoverlapping 95% CIs were considered to be indicative of significant between-subgroup differences, given the vital role of the 3-month mRS score in predicting long-term poststroke outcomes [30]. A meta-regression was performed on the basis of the patients’ mean age, with a *p* value of <0.05 indicating significance. A sensitivity analysis was performed for the 3-month mRS score by sequentially removing 1 study at a time to determine whether the overall effect was driven by a single study. To investigate the effects of OADs other than metformin on functional outcomes and neurological severity after stroke, we performed additional sensitivity analyses for studies comparing metformin monotherapy with other OAD regimens and those comparing OAD users with OAD nonusers. All analyses were performed using the Comprehensive Meta-Analysis software (version 3; Biostat, Englewood, NJ, USA).

### 2.8. Certainty of Evidence Assessment

The certainty of evidence for the primary outcome was assessed using the Grading of Recommendations Assessment, Development and Evaluation methodology. The initial rating was low because the sample did not include randomized controlled trials. The final rating was determined on the basis of the overall risk of bias, imprecision, inconsistency, indirectness, and publication bias [31].

## 3. Results

### 3.1. Study Selection and Characteristics

The initial search returned 47 potentially eligible articles. Of these articles, 11 [32,33,34,35,36,37,38,39,40,41,42] met our inclusion criteria (Figure 1). All eligible studies were cohort studies. A summary of the studies included in this review is presented in Table 2.

All 11 studies reported more favorable mRS scores for metformin users (metformin group) than for nonusers (nonmetformin group). However, 2 studies [35,42] revealed no significant between-group differences. Notably, 6 studies [32,34,35,36,37,40] reported mRS scores measured at 3 months after stroke onset, 5 studies [32,33,38,41,42] reported mRS scores measured at discharge, and 1 study [39] reported mRS scores measured at 12 months after stroke onset.

All studies reported lower NIHSS scores for the metformin group than for the nonmetformin group [32,33,34,35,36,37,38,39,40,41,42], with 7 studies [32,33,36,38,39,40,41] presenting significant results. In addition, 10 studies [32,33,35,36,37,38,39,40,41,42] reported NIHSS scores measured at admission, whereas 2 studies [32,34] reported NIHSS scores measured at discharge.

Four studies [32,34,37,40] reported poststroke sICH events. Of these studies, 2 [34,37] indicated significantly fewer sICH events in the metformin group than in the nonmetformin group (*p* < 0.009 [34]; *p* = 0.01 [30]). A total of 3 studies [32,34,40] reported sICH after IVT, whereas 2 [34,37] reported sICH after endovascular thrombectomy (EVT).

Five studies [32,34,39,40,41] reported lower rates of poststroke mortality in the metformin group than in the nonmetformin group. However, 1 study [39] observed no significant between-group difference. In addition, 2 studies [39,41] reported mortality during hospitalization, whereas 3 [32,34,40] reported mortality within 3 months after stroke onset.

Two studies [36,37] performed sensitivity analyses comparing the 3-month mRS score between patients who received metformin monotherapy and those who received other OADs, metformin with other OADs, or no OADs. Of these 2 studies, 1 study [37] reported that the 3-month mRS score significantly improved in the metformin monotherapy group.

Two studies [36,38] performed sensitivity analyses comparing NIHSS scores at admission between OAD users and nonusers. Of these, 1 study [38] reported significantly poorer NIHSS scores at admission in nonusers.

One study [37] analyzed the association between metformin dosage and functional outcomes. Kim et al. categorized metformin users into 3 dosage groups: <1000, 1000 to 2000, and >2000 mg/day. Only the 1000 to 2000 mg/day group exhibited functional outcomes that were significantly better (3-month mRS score: 0 to 2; OR: 4.17; 95% CI: 1.67 to 10.40) than those observed in the nonmetformin group.

Most studies focused on prestroke metformin use. Only 1 study [35] investigated the effect of poststroke metformin use. Jian et al. categorized metformin users into 3 groups: those who used metformin both before and after stroke onset (n = 94), those who stopped taking it after stroke onset (n = 30), and those who started taking it after stroke onset (n = 55). Only the group that used metformin both before and after stroke onset exhibited functional outcomes better than those observed in the nonmetformin group (proportion of patients with a 3-month mRS score of 0 to 1: 78.7% in the metformin group and 61.5% in the nonmetformin group; *p* = 0.006).

### 3.2. Risk of Bias

Of the 11 included studies, 5 [32,36,38,39,42] did not report patients’ renal function at stroke onset. To avoid lactic acidosis, metformin use is contraindicated in patients with CKD. Thus, renal function of the study participants may influence the use of metformin remarkably, and may confound the study results. Furthermore, 3 studies [36,39,41] did not report stroke subtypes. As an antidiabetic agent, metformin is much less likely to be used in patients with cardioembolic stroke, which is mostly associated with atrial fibrillation and valvular heart disease. If the composition of stroke subtype differed from the general population, the use of metformin would be confounded significantly. Hence, the aforementioned 6 studies [32,36,38,39,41,42] were assumed to have a high risk of bias because of the inadequate identification of confounding factors and an absence of strategies to address them. Further details are presented in Figure 2.

### 3.3. Functional Outcome (mRS Score)

#### 3.3.1. At Discharge

Four studies [32,33,38,41] reported higher crude ORs for favorable functional outcomes (mRS score: 0 to 2) in the metformin group than in the nonmetformin group. The pooled effect size for the crude data is presented in Figure 3 (OR: 1.184; 95% CI: 1.003 to 1.396; *p* = 0.046; *I*^2^ = 52.03%).

Two studies [33,38] used multivariate analyses to investigate the effect of metformin on the mRS score at discharge. The pooled effect size of the adjusted ORs indicated a higher likelihood of favorable functional outcomes in the metformin group (OR: 1.667; 95% CI: 0.933 to 2.979; *p* = 0.084; *I*^2^ = 0%; Figure 4) than in the nonmetformin group.

Allahverdiyev et al. [42] reported that the mean mRS score at discharge was lower in the metformin group than in the nonmetformin group (1.95 ± 1.14 vs. 2.54 ± 1.4); however, the between-group difference was nonsignificant (*p* = 0.697).

#### 3.3.2. At 3 Months After Stroke Onset

Four studies [32,35,36,40] reported higher crude ORs for favorable functional outcomes in the metformin group than in the nonmetformin group. The pooled effect size for the crude data is presented in Figure 5 (OR: 1.536; 95% CI: 1.231 to 1.915; *p* < 0.001; *I*^2^ = 67.56%). Among these studies, 2 [36,40] were multicenter studies, whereas the remaining 2 [32,35] were single-center studies. A subgroup analysis was performed on the basis of this difference. The pooled effect size for the multicenter studies suggested a significantly higher likelihood of favorable functional outcomes in the metformin group (OR: 1.756; 95% CI: 1.503 to 2.052; *p* < 0.001; *I*^2^ = 0%; Figure 6) than in the nonmetformin group. Similarly, the pooled effect size for the single-center studies indicated a significantly higher likelihood of favorable functional outcomes in the metformin group (OR: 1.256; 95% CI: 1.068 to 1.477; *p* = 0.006; *I*^2^ = 0%; Figure 7) than in the nonmetformin group. No significant difference was noted between the multicenter and single-center studies in the likelihood of favorable functional outcomes.

Three studies [35,36,37] used multivariate analyses to investigate the effect of metformin on the 3-month mRS score. The pooled effect size for the adjusted ORs indicated a higher likelihood of favorable functional outcomes in the metformin group (OR: 1.726; 95% CI: 1.027 to 2.901; *p* = 0.039; *I*^2^ = 62.1%; Figure 8) than in the nonmetformin group. Subgroup analysis could not be performed because of the limited number of relevant studies.

Curro et al. [34] reported significantly (*p* = 0.020) lower median 3-month mRS score in the metformin group than in the nonmetformin group (median [IQR]: 3 [1 to 5] vs. 4 [2 to 6]).

#### 3.3.3. At 12 Months After Stroke Onset

Tu et al. [39] reported mRS scores. They identified significantly (*p* < 0.001) lower odds of “case disability” (mRS score: 3 to 5) in the metformin group than in the nonmetformin group (14.74% [95% CI: 13.51 to 15.98] vs. 19.41% [95% CI: 18.06 to 20.76]).

### 3.4. Neurological Severity (NIHSS Score)

#### 3.4.1. At Admission

Five studies [32,33,36,37,38] reported higher crude ORs for mild stroke at admission in the metformin group than in the nonmetformin group. However, the definition of “mild stroke” varied across the included studies. Three studies [32,36,37] defined mild stroke as an NIHSS score of 0 to 4, 1 study [33] defined it as an NIHSS score of 0 to 3, and another study [38] defined it as an NIHSS score of 0 to 2. The pooled effect size for the crude data is presented in Figure 9 (OR: 1.605; 95% CI: 1.100 to 2.343; *p* = 0.014; *I*^2^ = 74.98%).

Three studies [33,36,38] used multivariate analyses to investigate the effect of metformin on the risk of mild stroke at admission. The pooled effect size for the adjusted ORs indicated a higher risk of mild stroke in the metformin group (OR: 1.645; 95% CI: 1.278 to 2.116; *p* < 0.001; *I*^2^ = 0%; Figure 10) than in the nonmetformin group.

Eight studies [32,33,35,37,39,40,41,42] reported lower NIHSS scores at admission in the metformin group than in the nonmetformin group. The pooled effect size is presented in Figure 11 (SMD: −0.138; 95% CI: −0.230 to −0.046; *p* = 0.003; *I*^2^ = 76.03%).

Two studies [40,41] used multivariate analyses to investigate the effect of metformin on NIHSS scores at admission. The pooled effect size indicated lower NIHSS scores in the metformin group (SMD: −0.187; 95% CI: −0.267 to −0.107; *p* < 0.001; *I*^2^ = 0%; Figure 12) than in the nonmetformin group.

#### 3.4.2. At Discharge

Two studies [32,34] reported lower NIHSS scores at discharge in the metformin group than in the nonmetformin group. The pooled effect size is presented in Figure 13 (SMD: −0.09; 95% CI: −0.171 to −0.008; *p* = 0.031; *I*^2^ = 0%).

### 3.5. sICH

Three studies [32,34,40] reported post-IVT sICH events in the metformin and nonmetformin groups. The pooled effect size is presented in Figure 14 (OR: 0.989; 95% CI: 0.440 to 2.221; *p* = 0.979; *I*^2^ = 41.17%).

Currò et al. [34] reported a higher rate of post-EVT sICH events in the metformin group than in the nonmetformin group (4 [9.3%] vs. 3 [6.12%]; *p* = 0.566).

### 3.6. Mortality

Three studies [32,34,40] reported lower risks of mortality at 3 months after stroke onset in the metformin group than in the nonmetformin group. The pooled effect size is presented in Figure 15 (OR: 0.635; 95% CI: 0.511 to 0.789; *p* < 0.001; *I*^2^ = 0%).

Two studies [39,41] reported lower risks of mortality before discharge in the metformin group than in the nonmetformin group. The pooled effect size is presented in Figure 16 (OR: 0.686; 95% CI: 0.338 to 1.391; *p* = 0.296; *I*^2^ = 92.88%).

### 3.7. Results of Sensitivity Analysis

In the sensitivity analysis, the OR for 3-month favorable functional outcomes was the lowest (1.429; 95% CI: 1.130 to 1.806) when the study by Kersten et al. [36] was excluded and the highest (1.717; 95% CI: 1.479 to 1.993) when that by Akhtar et al. [32] was excluded. In the sensitivity analysis of metformin monotherapy versus other regimens, 2 studies [36,37] used multivariate analyses to investigate the effect of metformin monotherapy on the 3-month mRS score. The pooled effect size for adjusted ORs indicated a higher likelihood of favorable functional outcomes in the metformin monotherapy group (OR: 1.693; 95% CI: 0.703 to 4.082; *p* = 0.241; *I*^2^ = 74.03%; Figure 17) than in the other group.

In the sensitivity analysis of OAD users versus nonusers, 2 studies [36,38] used multivariate analyses to investigate the effect of no OAD use on NIHSS at admission. The pooled effect size for adjusted ORs indicated a lower risk of mild stroke in OAD nonusers (OR: 0.618; 95% CI: 0.370 to 1.034; *p* = 0.067; *I*^2^ = 66.7%; Figure 18) than in OAD users.

### 3.8. Results of Meta-Regression

Because of the limited number of studies using multivariate analyses to investigate the effect of metformin on favorable functional outcomes, we performed a meta-regression on the basis of crude ORs for favorable functional outcomes at 3 months after stroke onset. The analysis revealed a significant effect of age on functional outcome (*p* = 0.003), indicating that the difference between the metformin and nonmetformin groups in the likelihood of favorable functional outcomes was larger in studies with older participants.

### 3.9. Publication Bias

We detected possible publication bias for the crude ORs for favorable functional outcomes at 3 months after stroke onset, as indicated by visual asymmetry on the funnel plot presented in Figure 19. Similarly, possible publication bias was noted in the adjusted ORs, as highlighted by the funnel plot presented in Figure 20.

### 3.10. Certainty of Evidence

The certainty of evidence for the effect of metformin on favorable functional outcomes at 3 months after stroke onset was rated very low. The initial rating was low because the sample included no randomized controlled trials. Moreover, because of considerable inconsistency between the metformin and nonmetformin groups, the rating further declined to very low (Table 3).

The certainty of evidence for NIHSS at admission and post-IVT sICH were also rated very low. The initial ratings were low because the sample included no randomized controlled trials. The ratings declined to very low due to considerable inconsistency between groups and considerable imprecision of pooled effect, respectively (Table 4 and Table 5).

The certainty of evidence for mortality at 3 months after stroke onset was rated low. The initial rating was low because the sample included no randomized controlled trials (Table 6).

## 4. Discussion

Among the patients with acute ischemic stroke and T2DM, metformin users had lower stroke severity (measured using the NIHSS) at admission and better functional outcomes (measured using the mRS) at 3 months after stroke onset than the nonmetformin users. Consistent with research assessing the effect of metformin on functional recovery in an experimental model [16], Pakkam et al. [20] reported a higher likelihood of favorable functional outcomes at discharge in the metformin group than in the nonmetformin group. Elgenidy et al. [21] also reported a higher likelihood of favorable poststroke functional outcomes in the metformin group than in the nonmetformin group. However, the researchers pooled the mRS score at discharge and that at 3 months after stroke, which may reduce the reliability of their conclusion. Consistent with the literature, Mohammadi et al. [22] reported that the metformin group had a higher likelihood of favorable functional outcomes at 90 days after stroke onset than the nonmetformin group. These findings align with ours. We performed a meta-analysis of adjusted ORs for favorable functional outcomes, which increased the reliability of results pertaining to metformin’s effects on functional outcomes. Two studies [21,22] reported lower NIHSS scores at admission in the metformin group than in the nonmetformin group. This finding is corroborated by our results indicating a significantly higher risk (both crude and adjusted ORs) of mild stroke in the metformin group than in the nonmetformin group. The result aligns with those of animal studies [14,15]. Furthermore, consistent with relevant reviews [20,22], our meta-analysis revealed similar rates of sICH between the metformin and nonmetformin groups. However, the rate of mortality was lower in the metformin group than in the nonmetformin group. The reduced mortality rate may be attributable to metformin’s effects against neurologic problems [14,15] and other diseases [10,11]. However, caution should be exercised when interpreting these findings because the results were derived from crude comparisons and were prone to confounding.

In sensitivity analyses, metformin monotherapy was associated with favorable functional outcomes. The severity of stroke was higher in patients with T2DM not using OADs than in those using OADs. These associations support metformin’s neuroprotective effect. However, the small sample size in this study limited the statistical power of our meta-analysis. Jian et al. [35] performed a sensitivity analysis of functional outcomes on the basis of the timing of metformin initiation. The researchers noted a significantly higher likelihood of favorable functional outcomes in patients using metformin before and after stroke onset than in those not using this drug (proportion of patients with a 3-month mRS score of 0 to 1: 78.7% vs. 61.5%, respectively; *p* = 0.006). Furthermore, the likelihood of a favorable functional outcome was higher in patients who started using metformin after stroke onset than in nonusers, but the result was nonsignificant (n = 55; proportion of patients with an mRS score of 0 to 1: 76.4%; *p* = 0.052), likely because of insufficient statistical power. These findings indicate that metformin still exerts a neuroprotective effect when it is initiated after stroke onset, although this warrants further research.

Kim et al. [37] performed a sensitivity analysis of functional outcomes on the basis of metformin dosage. They found that compared with their nonmetformin group, the 1000 to 2000 mg/day group—but not the <1000 or >2000 mg/day group—was more likely to achieve favorable functional outcomes after stroke onset. This finding indicates that the neuroprotective effect of metformin follows a dome-shaped pattern, rather than a dose-dependent pattern, differing from the finding of an animal study [43]. Further studies are required to validate the association between metformin dosage and functional outcomes.

A large-scale cohort study of 14,558 patients with T2DM [44] revealed a significant association between metformin’s protective effect against neurodegenerative diseases and its cumulative dosage. An animal study [43] highlighted a dose-dependent protective effect. Future research should confirm whether cumulative metformin dosage influences its protective effect against ischemic stroke.

Our meta-regression highlighted larger differences in the likelihood of favorable functional outcomes between the metformin and nonmetformin groups in studies with older participants (*p* = 0.003). This finding may be attributable to the protective effects of metformin against cardiovascular diseases [10,11] and neurodegenerative diseases [12], given that older adults are more vulnerable to these comorbidities. However, because our meta-regression was based on crude ORs for favorable functional outcomes, this result was prone to confounding.

Although the Egger test result for our primary outcome (3-month mRS score) did not reach significance (*p* = 0.132), visual inspection of the funnel plot revealed asymmetry. Therefore, the Egger test might have yielded a false-negative result because of insufficient statistical power resulting from the small sample size [45]. The funnel plot indicated that studies with higher standard errors (fewer participants) reported higher ORs, likely reflecting journals’ tendency to avoid publishing small-scale studies with low statistical power and nonsignificant results. During article selection, we identified a study with 59 patients with T2DM with acute ischemic stroke [42], wherein no significant difference in poststroke mRS score was observed between the metformin and nonmetformin groups. The aforementioned study was excluded from our meta-analysis because of inadequate outcome data. Although our study highlights a promising neuroprotective effect of metformin, the high risk of publication bias should be considered in interpreting the results.

The present study has several strengths. To the best of our knowledge, this study is the first meta-analysis to explore the following aspects. First, this is the first meta-analysis to investigate the association between metformin use and neurological severity (measured using the NIHSS). Second, this is the first meta-analysis to estimate adjusted ORs for favorable functional outcomes (measured using the mRS) in metformin users. Third, this is the first meta-analysis to involve a sensitivity analysis of the effects of different OAD regimens (metformin monotherapy vs. OAD nonuse) on functional outcomes and neurological severity. Finally, this is the first meta-analysis to highlight publication bias on this topic.

Notably, our study has several limitations. First, the certainty of evidence was limited by the observational design of the included studies. Differences between the metformin and nonmetformin groups in age, sex, renal function, and serum glucose level at admission might have obscured the actual effect of metformin on poststroke functional outcomes. Second, nearly none of the included studies reported the duration of metformin use before stroke or whether it was continued after stroke. Only 1 study [35] investigated the effect of continued metformin use on functional outcomes. Further studies are required to determine the effective dose and duration of metformin for neuroprotective benefits. Third, poststroke reperfusion therapies varied across the included studies. One study [40] included only patients who underwent IVT after acute ischemic stroke onset, 1 study [37] included only those who underwent EVT, 1 study included those who underwent IVT or EVT [34], and 1 study [33] excluded those who underwent EVT. Five studies [32,35,36,39,41] included patients undergoing IVT, those undergoing EVT, and those not receiving reperfusion therapy. One study [38] did not report whether patients received reperfusion therapy. These inconsistencies might have influenced the functional outcomes. Fourth, there was high heterogeneity noted in some of our analysis (for example, *I*^2^ of 67.56% in favorable functional outcome at 3 months). Different poststroke reperfusion therapies may contribute largely to this finding. The diversity of patient characteristics (for example, the mean age of stroke onset varied from 54.4 to 76.7) and sample sizes (from 70 to 7587) across the included studies may also have played a role. Fifth, the definition of “mild stroke” at admission differed among the included studies (NIHSS 0–2, 0–3, 0–4). Given the limited number of studies, we pooled these data into a meta-analysis, which may have increased the heterogeneity across the included studies, and may have decreased the reliability of the results. Sixth, our search was restricted to English publications, and this may have introduced language bias.

In terms of clinical implications, our study provides valuable insights into the neuroprotective effect of metformin. This drug is already used as a first-line therapy for patients with T2DM without severe renal impairment [9], and its neuroprotective properties have been demonstrated [20]. Our study consolidated the real-world effect of metformin, highlighted the limitations of interpreting current evidence, and underscored the potential of metformin as a treatment for ischemic stroke.

We have several recommendations for future studies. First, future studies should recruit participants with homogeneous demographic characteristics. Because the selection of OADs is highly personalized, being influenced by factors such as hypoglycemia risk, renal function, cardiac function, and financial burden, conducting prospective studies may be challenging. Retrospective studies on this topic should consider propensity score matching to balance the baseline characteristics between patient groups. Second, future studies should include subgroup analyses based on metformin dose, duration, and continuation. Although current evidence highlights metformin’s potential as a treatment option for ischemic stroke, further research is required to clarity its effects across dosages, durations, and continuation patterns. Finally, future studies should investigate the effect of reperfusion therapy on the neuroprotective effect of metformin. Given the increasing importance of reperfusion therapies, particularly EVT, in the management of ischemic stroke, future studies should explore the interaction between reperfusion therapies and metformin’s effect on poststroke functional outcomes.

## 5. Conclusions

Among patients with T2DM with acute ischemic stroke, metformin use is associated with reduced stroke severity and improved functional outcomes at 3 months after stroke onset, which was observed in the crude and adjusted odds ratios. However, high heterogeneity of the included studies and very low certainty of evidence may temper the reliability of our findings. Randomized controlled trials are necessary to confirm the actual effects of metformin on stroke severity and long-term functional outcomes.

## Figures and Tables

**Figure 1:**
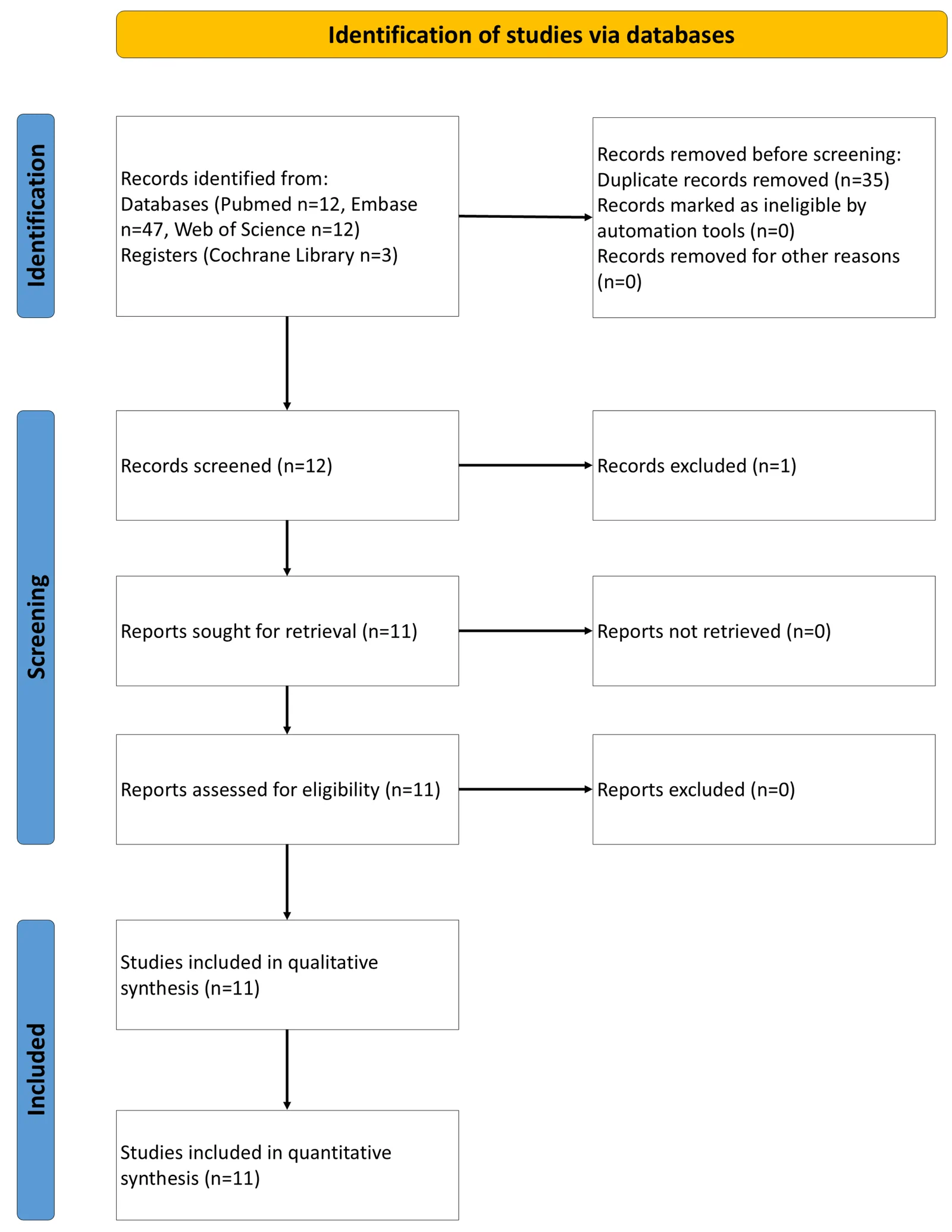
Flowchart depicting article selection.

**Figure 2:**
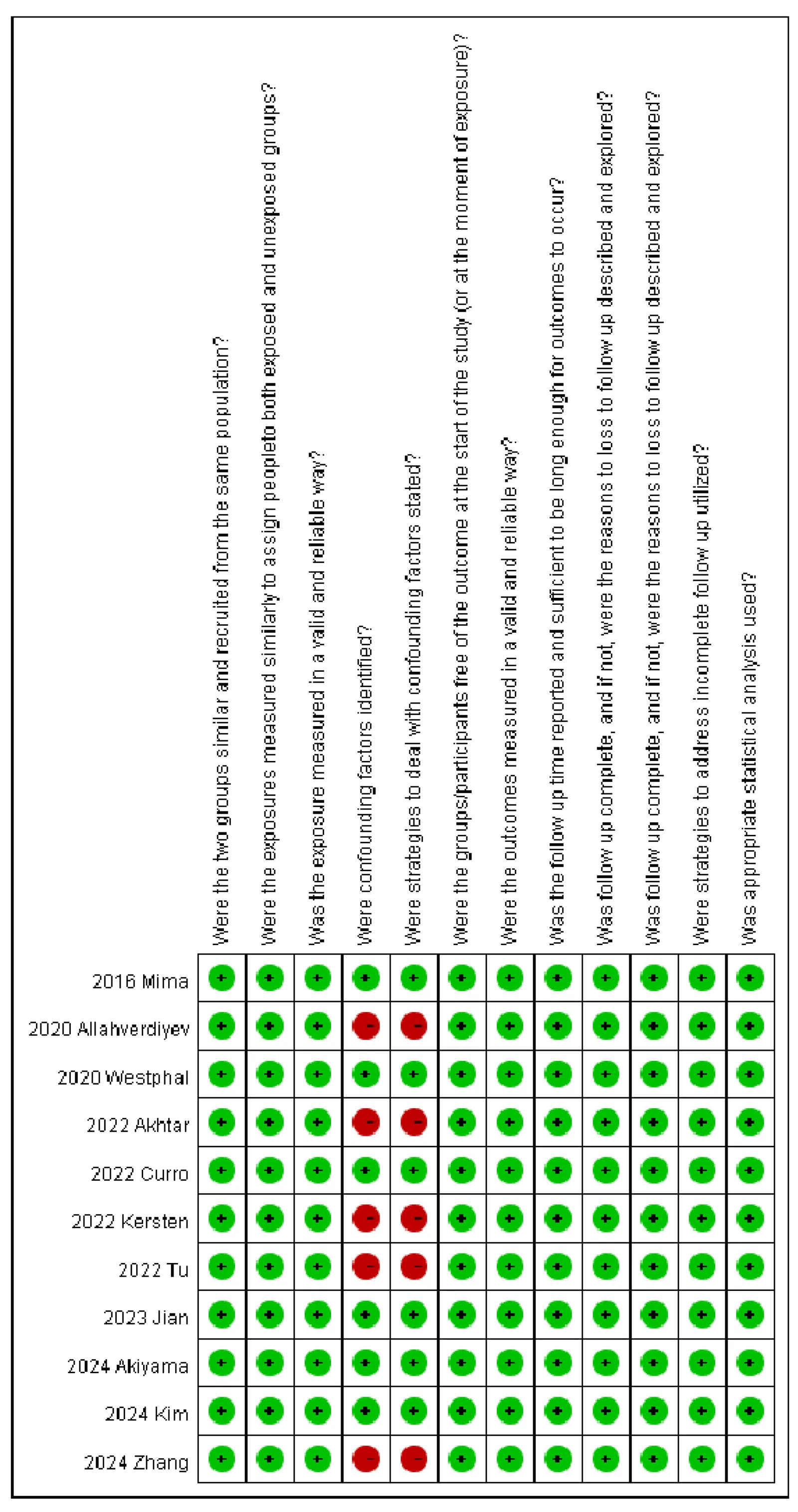
Summary table depicting risk of bias in the included studies [32,33,34,35,36,37,38,39,40,41,42].

**Figure 3:**
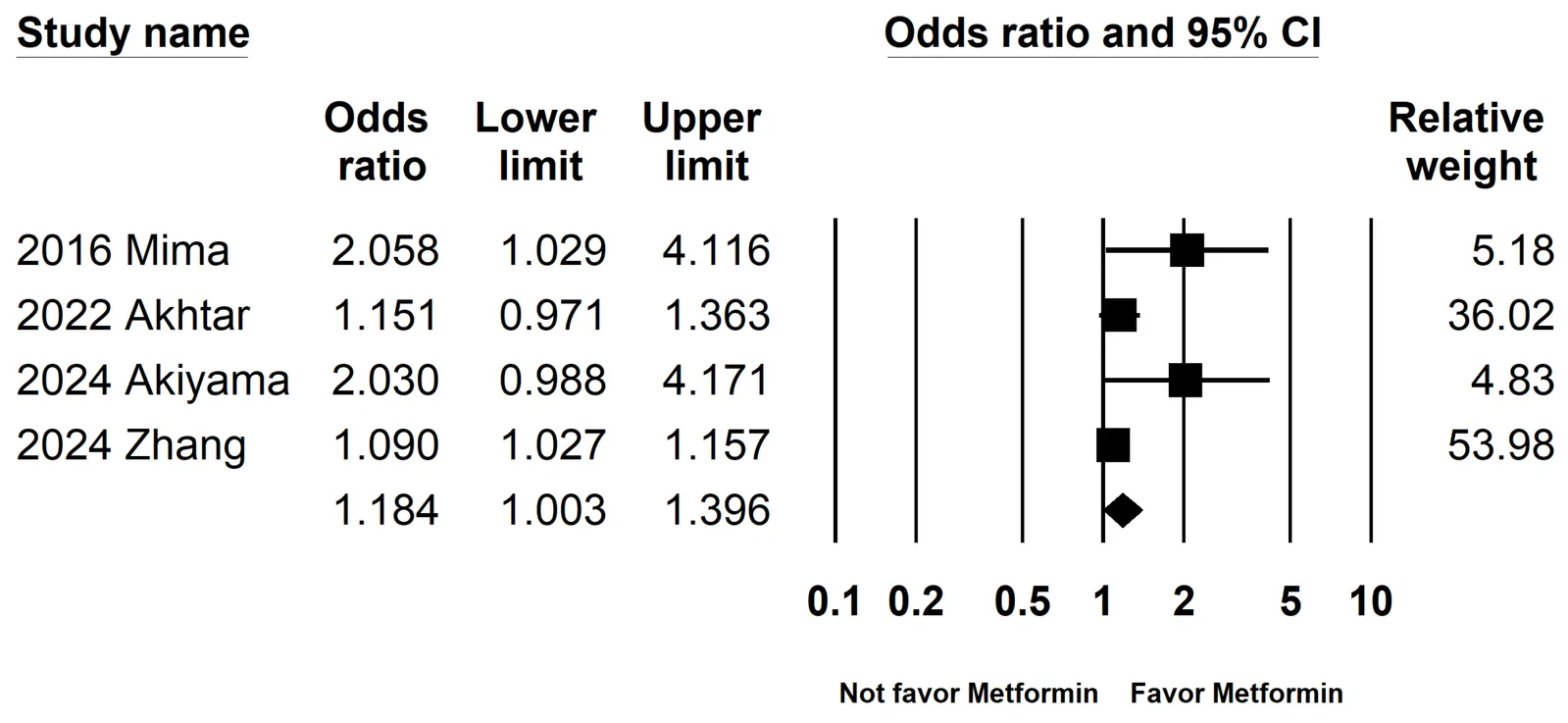
Forest plot depicting crude ORs for favorable functional outcomes at discharge [32,33,38,41].

**Figure 4:**
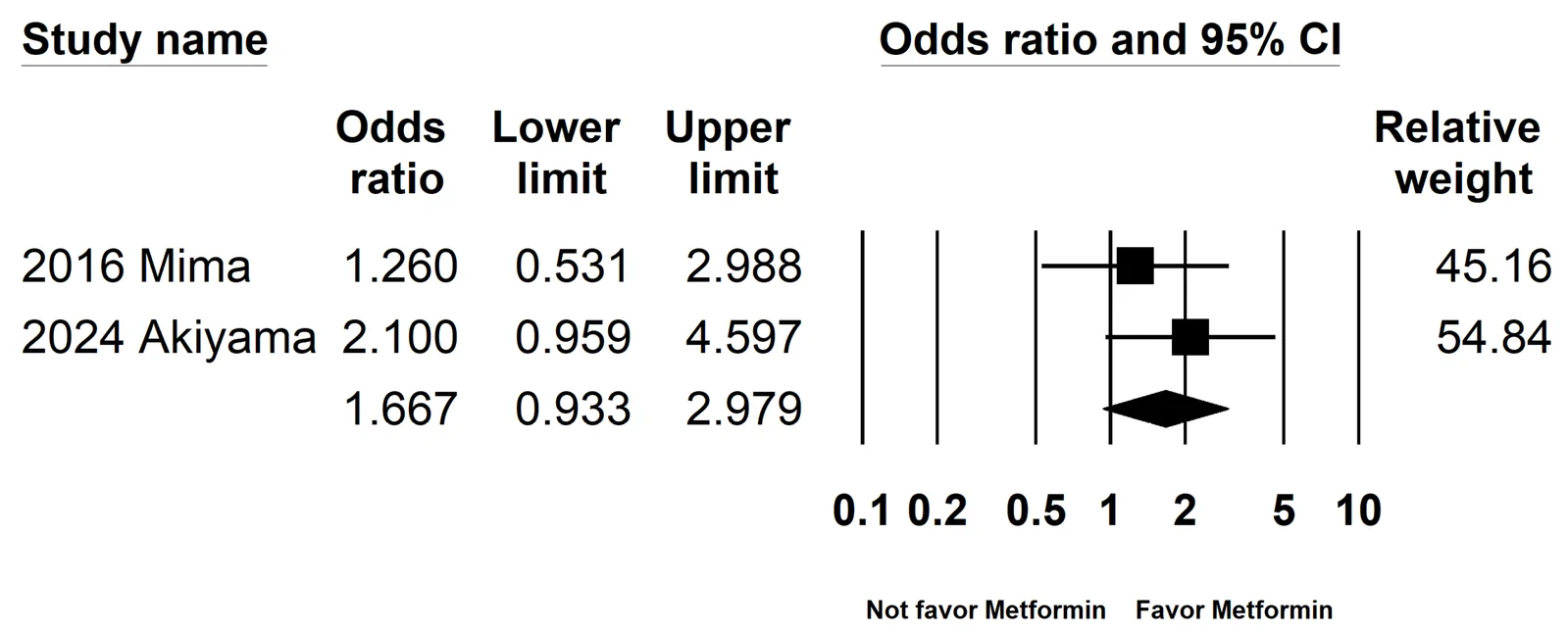
Forest plot depicting adjusted ORs for favorable functional outcomes at discharge [33,38].

**Figure 5:**
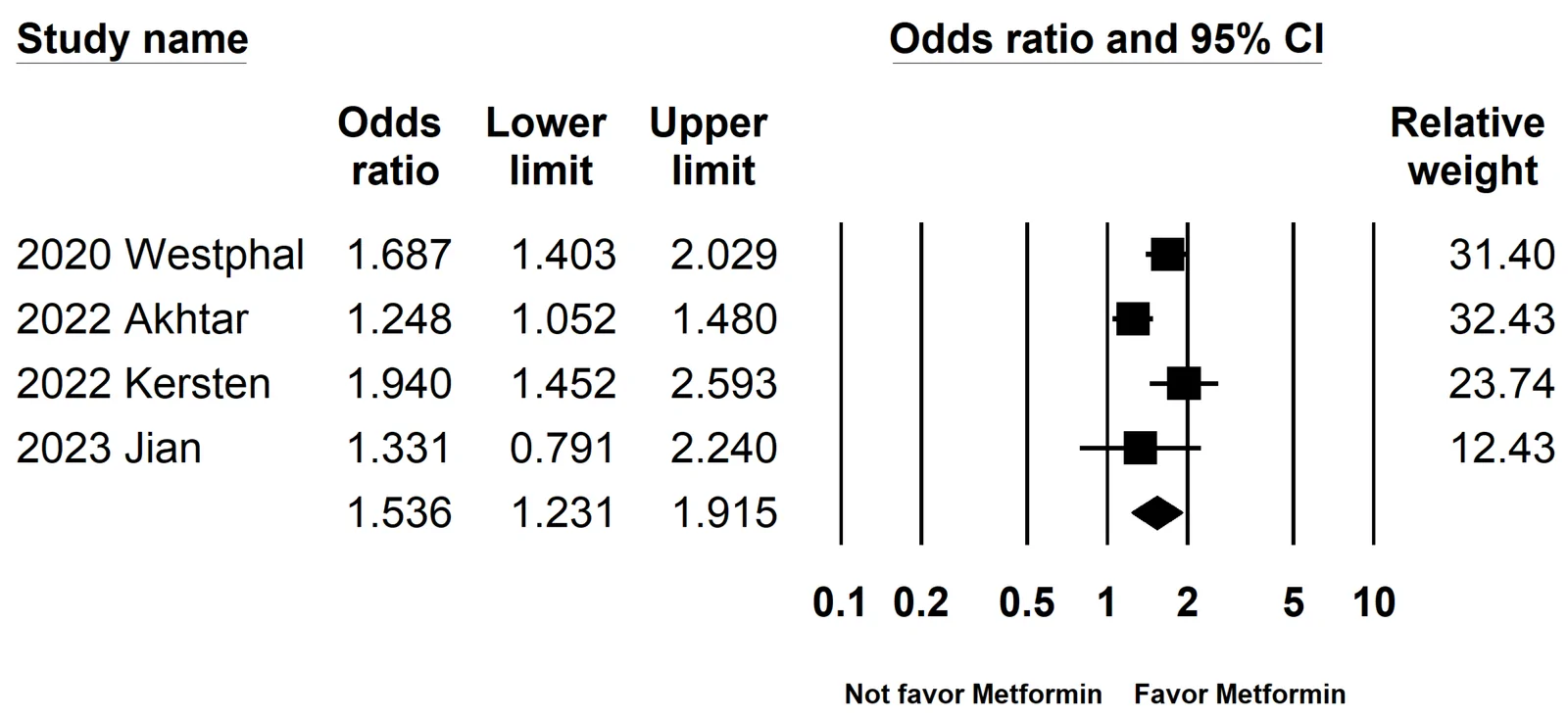
Forest plot depicting crude ORs for favorable functional outcomes at 3 months after stroke onset [32,35,36,40].

**Figure 6:**
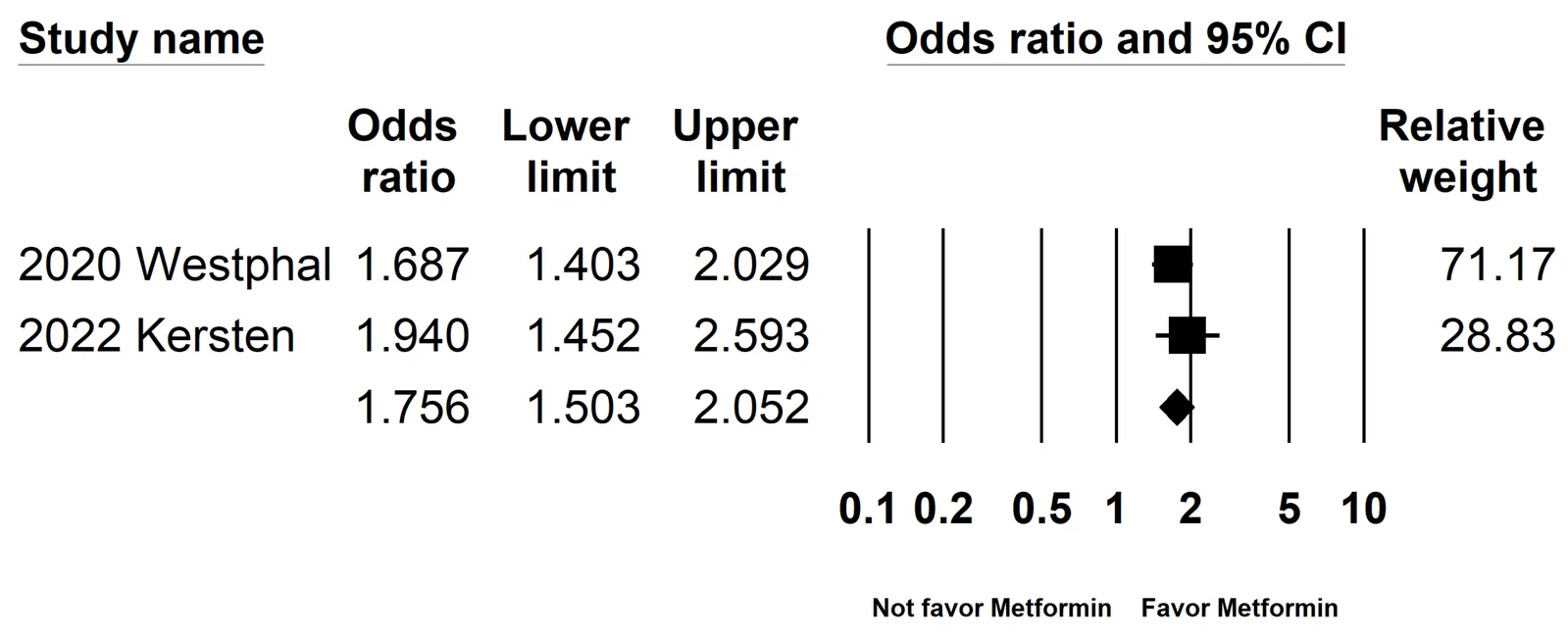
Forest plot depicting crude ORs for favorable functional outcomes at 3 months after stroke onset, multicenter studies [36,40].

**Figure 7:**
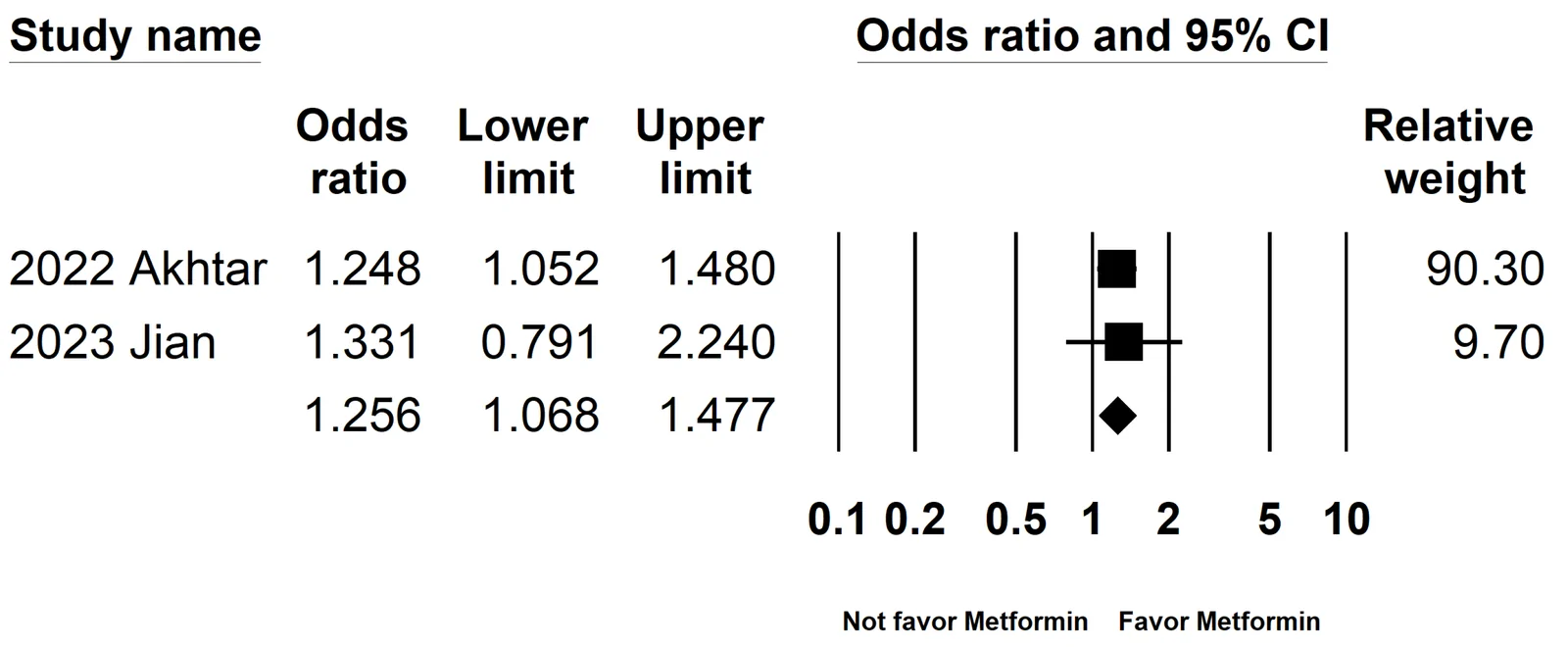
Forest plot depicting crude ORs for favorable functional outcomes at 3 months after stroke onset, single-center studies [32,35].

**Figure 8:**
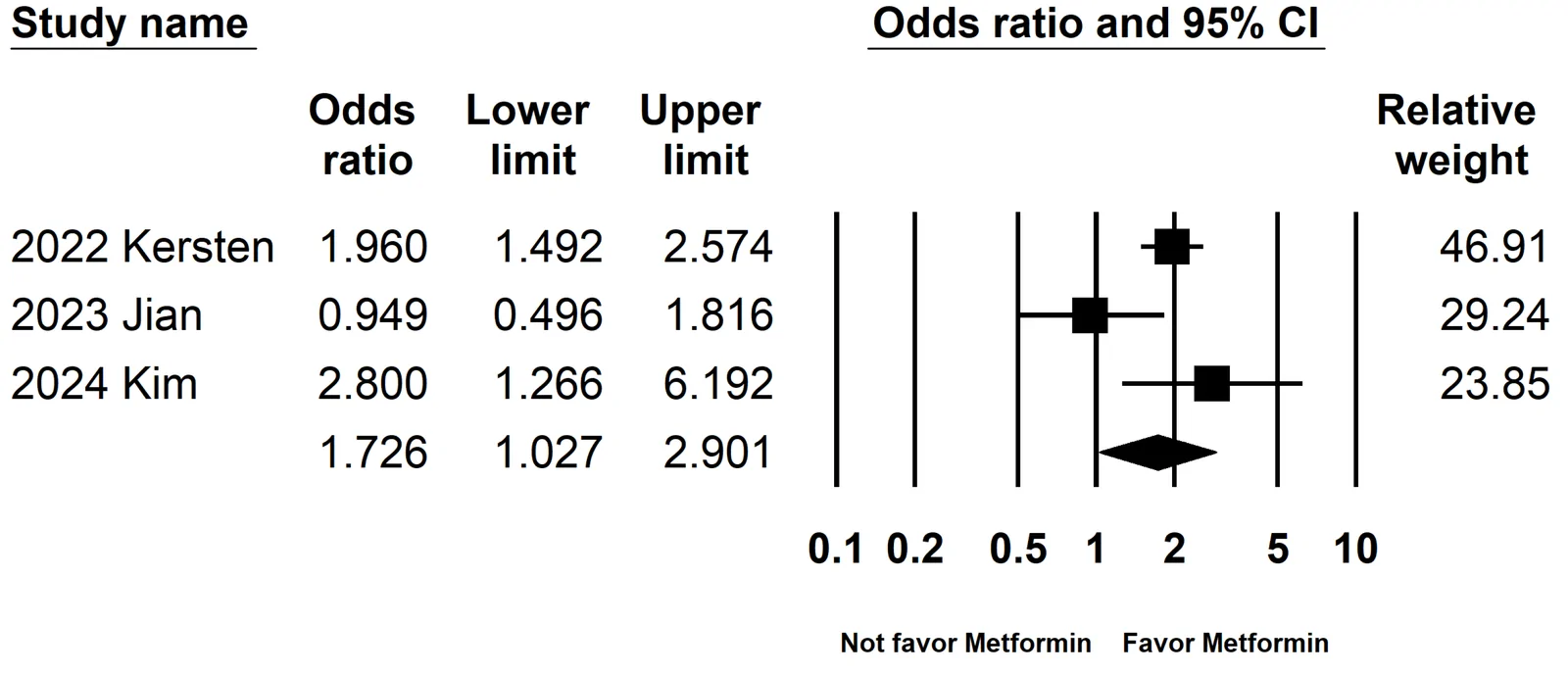
Forest plot depicting adjusted ORs for favorable functional outcomes at 3 months after stroke onset [35,36,37].

**Figure 9:**
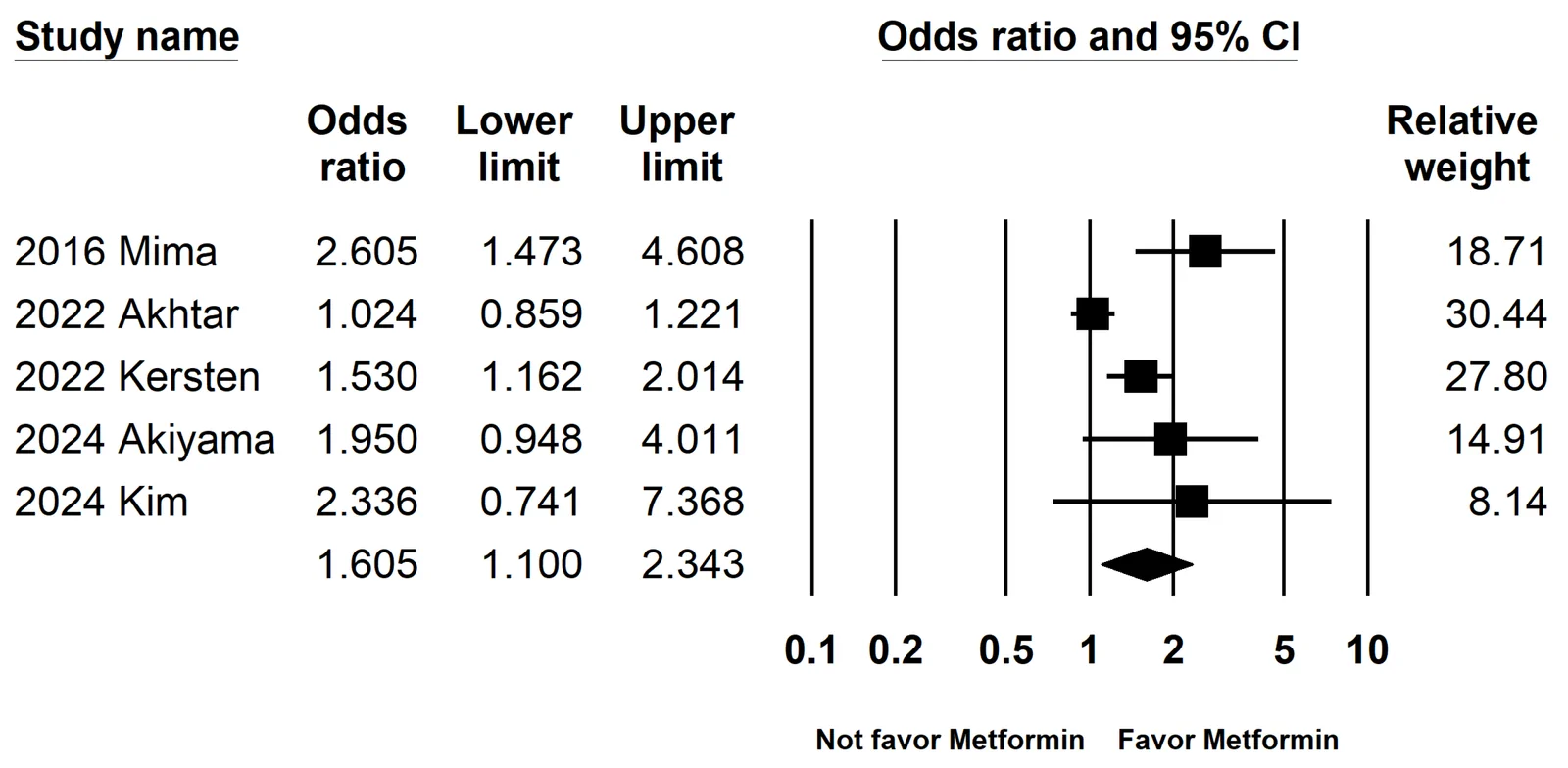
Forest plot depicting crude ORs for risk of mild stroke at admission [32,33,36,37,38].

**Figure 10:**
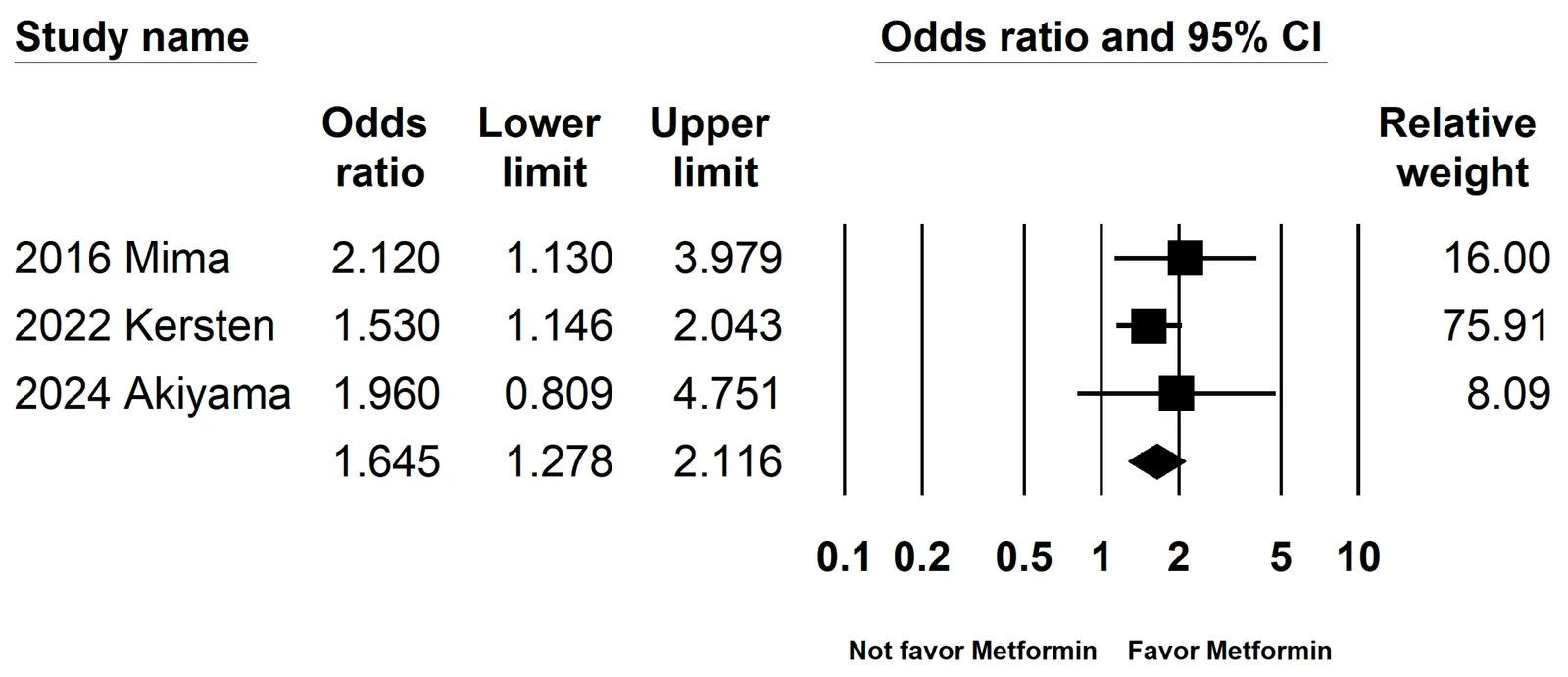
Forest plot depicting adjusted ORs for risk of mild stroke at admission [33,36,38].

**Figure 11:**
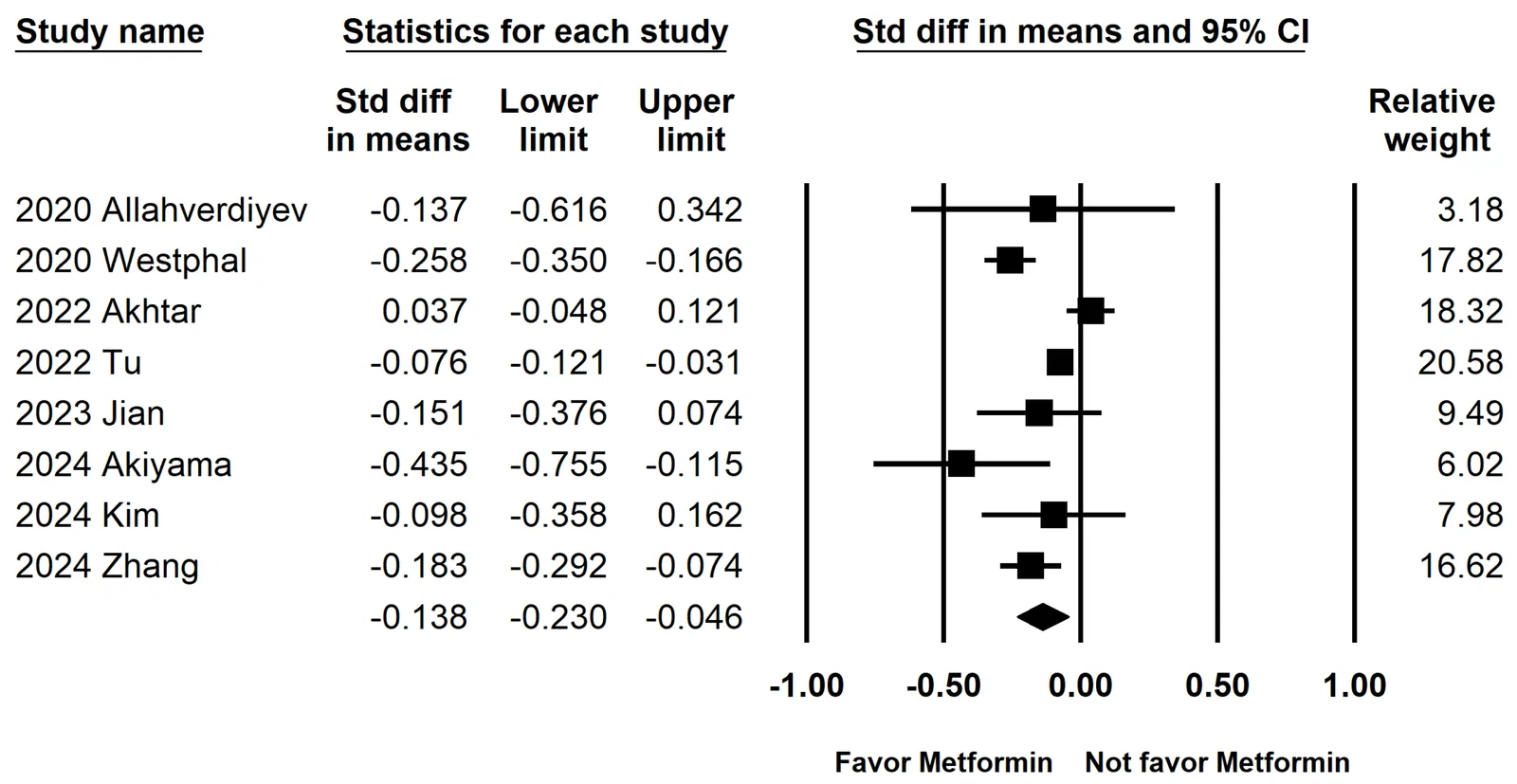
Forest plot depicting National Institutes of Health Stroke Scale scores at admission [32,33,35,37,39,40,41,42].

**Figure 12:**
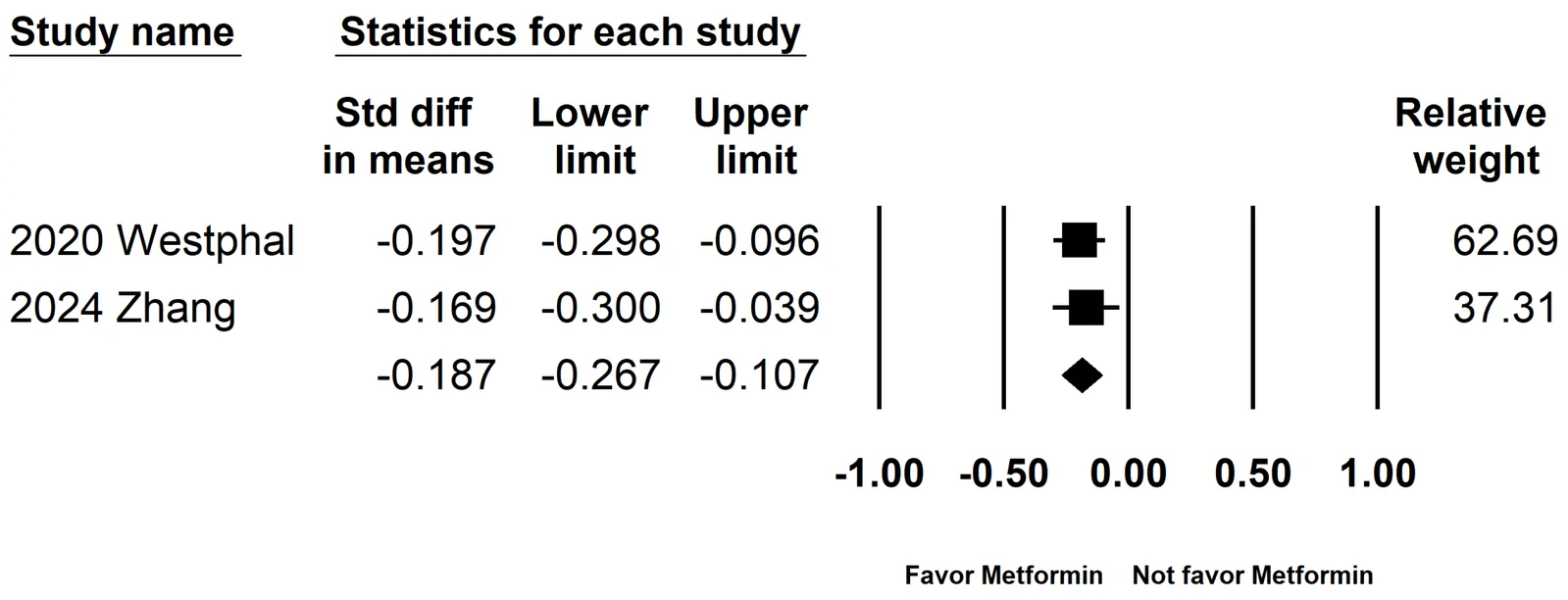
Forest plot depicting adjusted National Institutes of Health Stroke Scale scores at admission [36,41].

**Figure 13:**
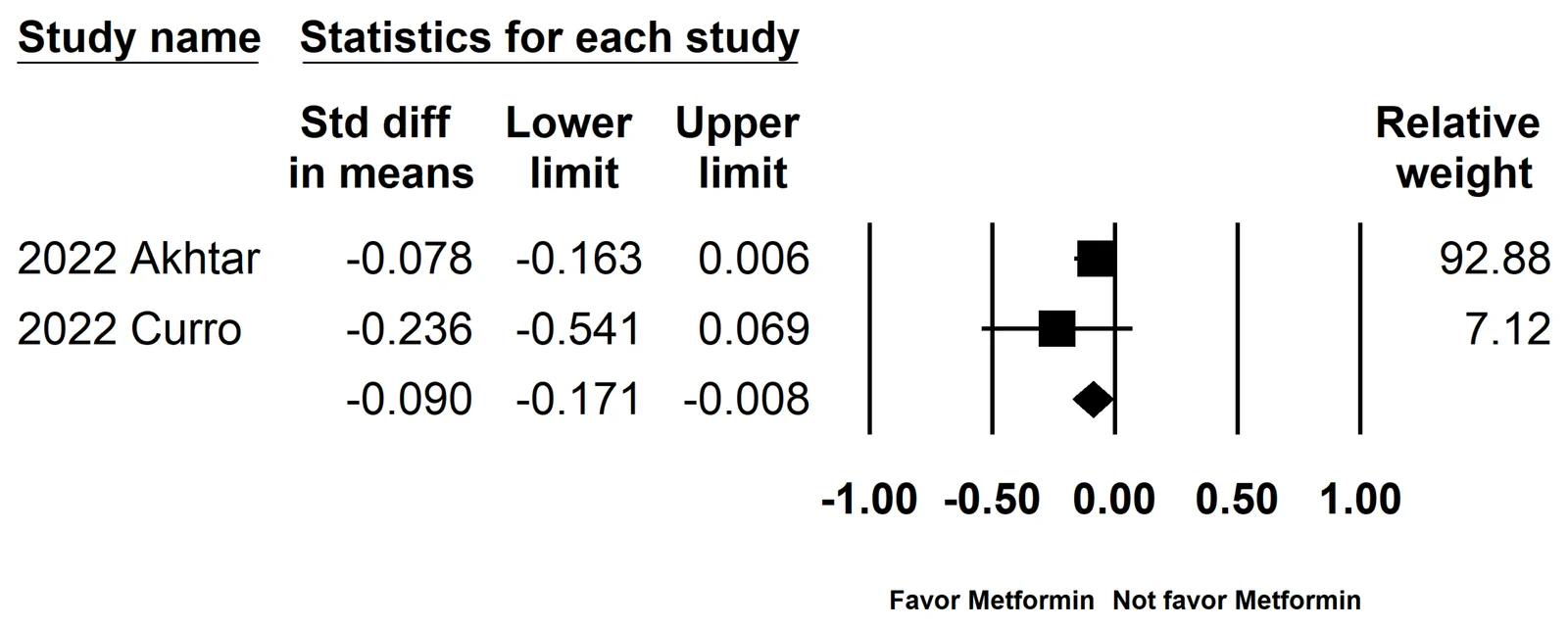
Forest plot depicting National Institutes of Health Stroke Scale scores at discharge [32,34].

**Figure 14:**
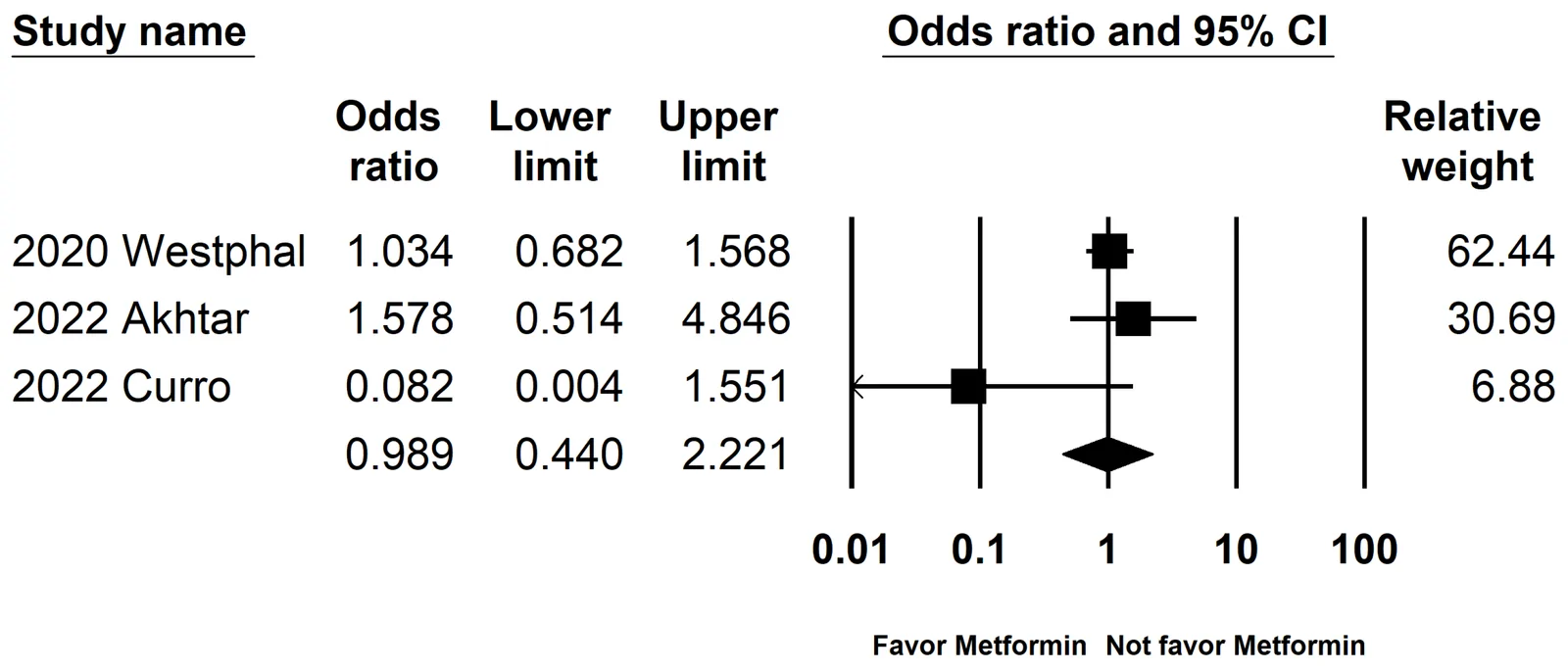
Forest plot depicting risk of symptomatic intracranial hemorrhage after intravenous thrombolysis [32,34,40].

**Figure 15:**
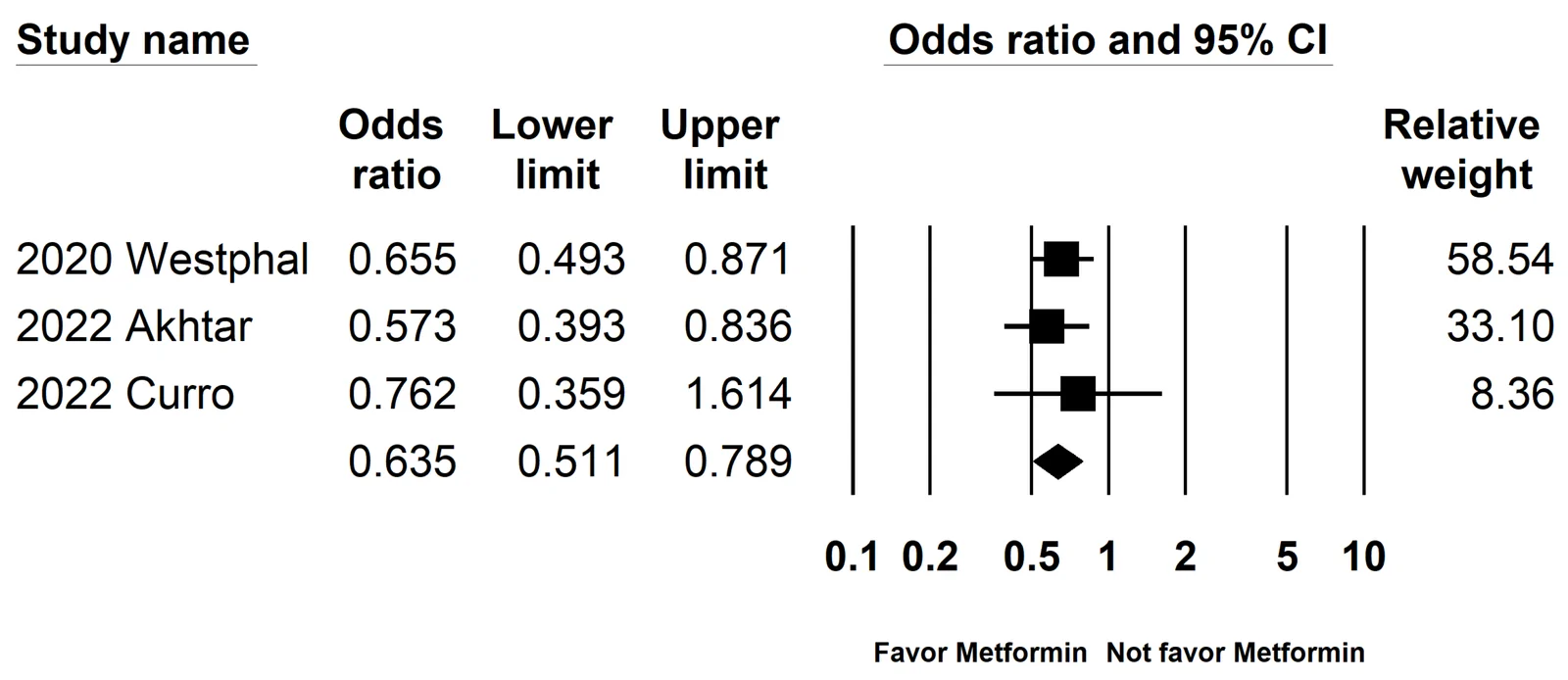
Forest plot depicting risk of mortality at 3 months after stroke onset [32,34,40].

**Figure 16:**
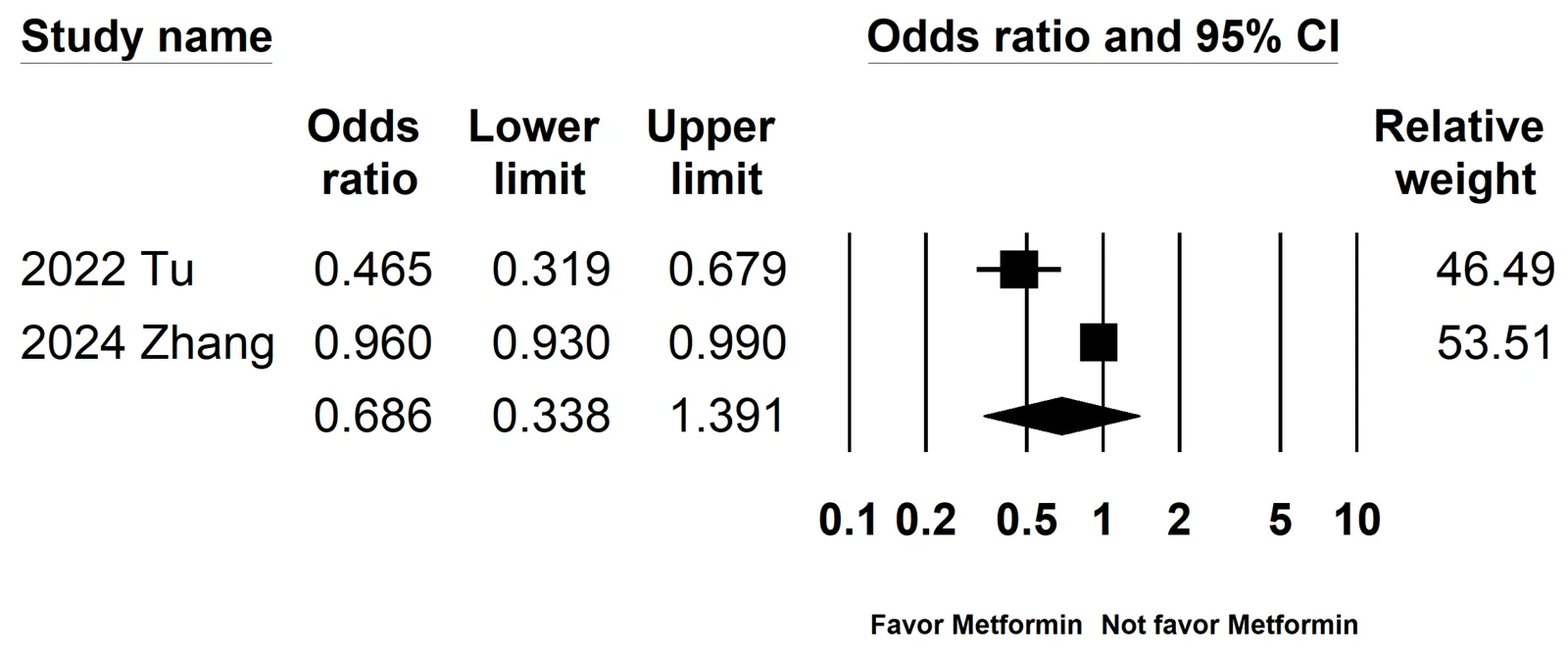
Forest plot depicting risk of mortality before discharge [39,41].

**Figure 17:**
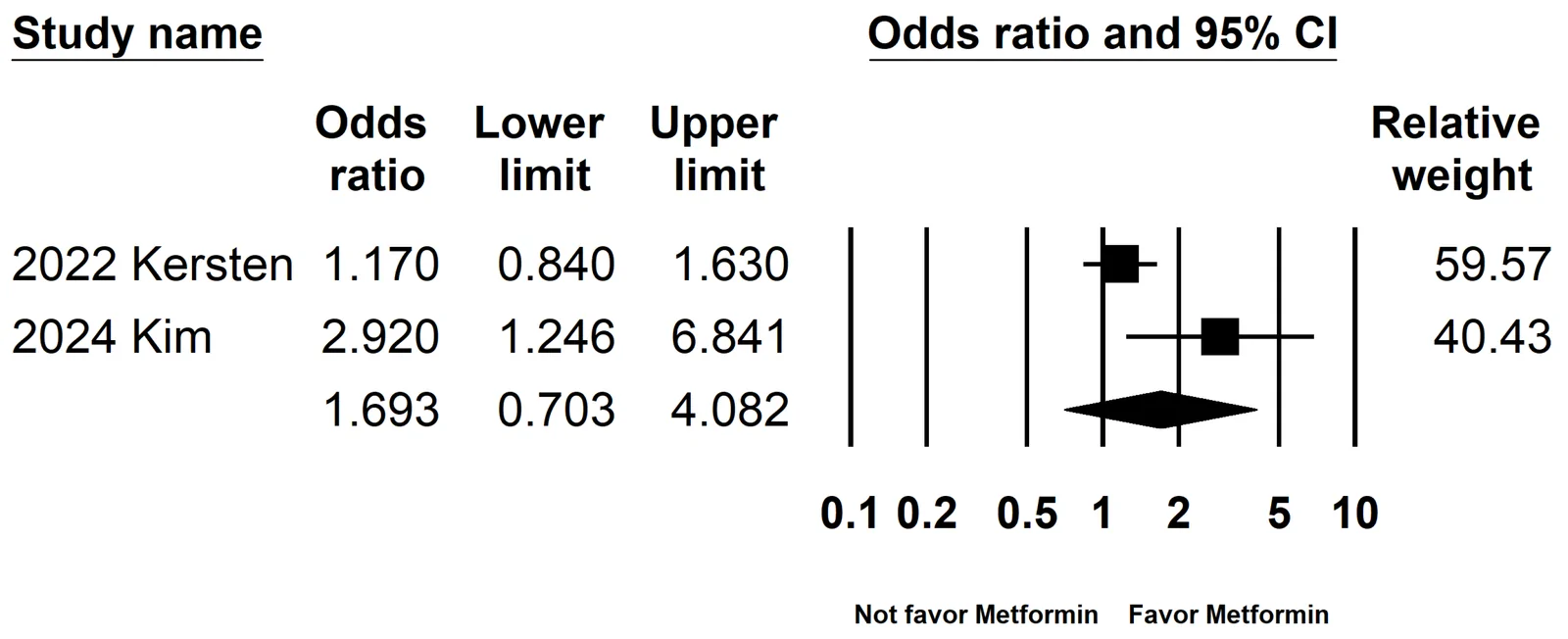
Forest plot depicting adjusted ORs for favorable functional outcomes at 3 months after stroke onset in patients receiving metformin monotherapy [36,37].

**Figure 18:**
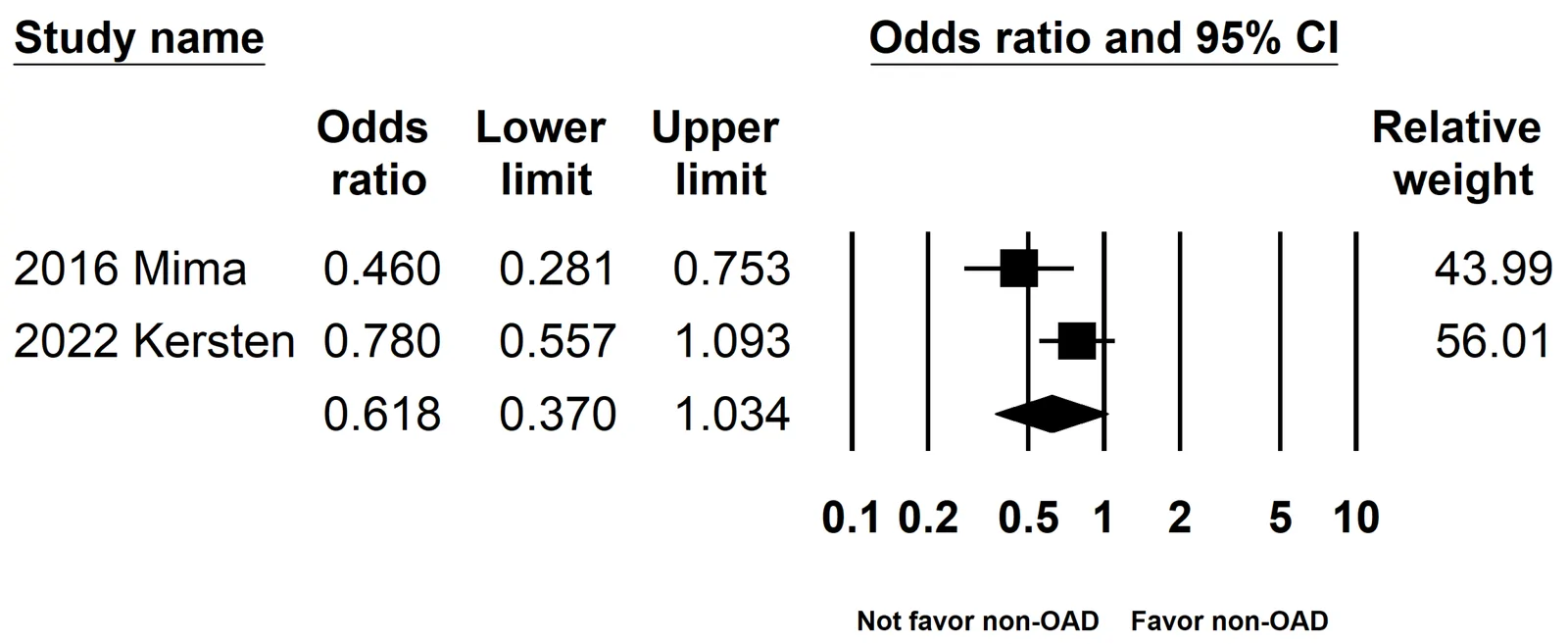
Forest plot depicting risk of mild stroke at admission in non-OAD users. Abbreviation: OAD, oral antidiabetic drug [36,38].

**Figure 19:**
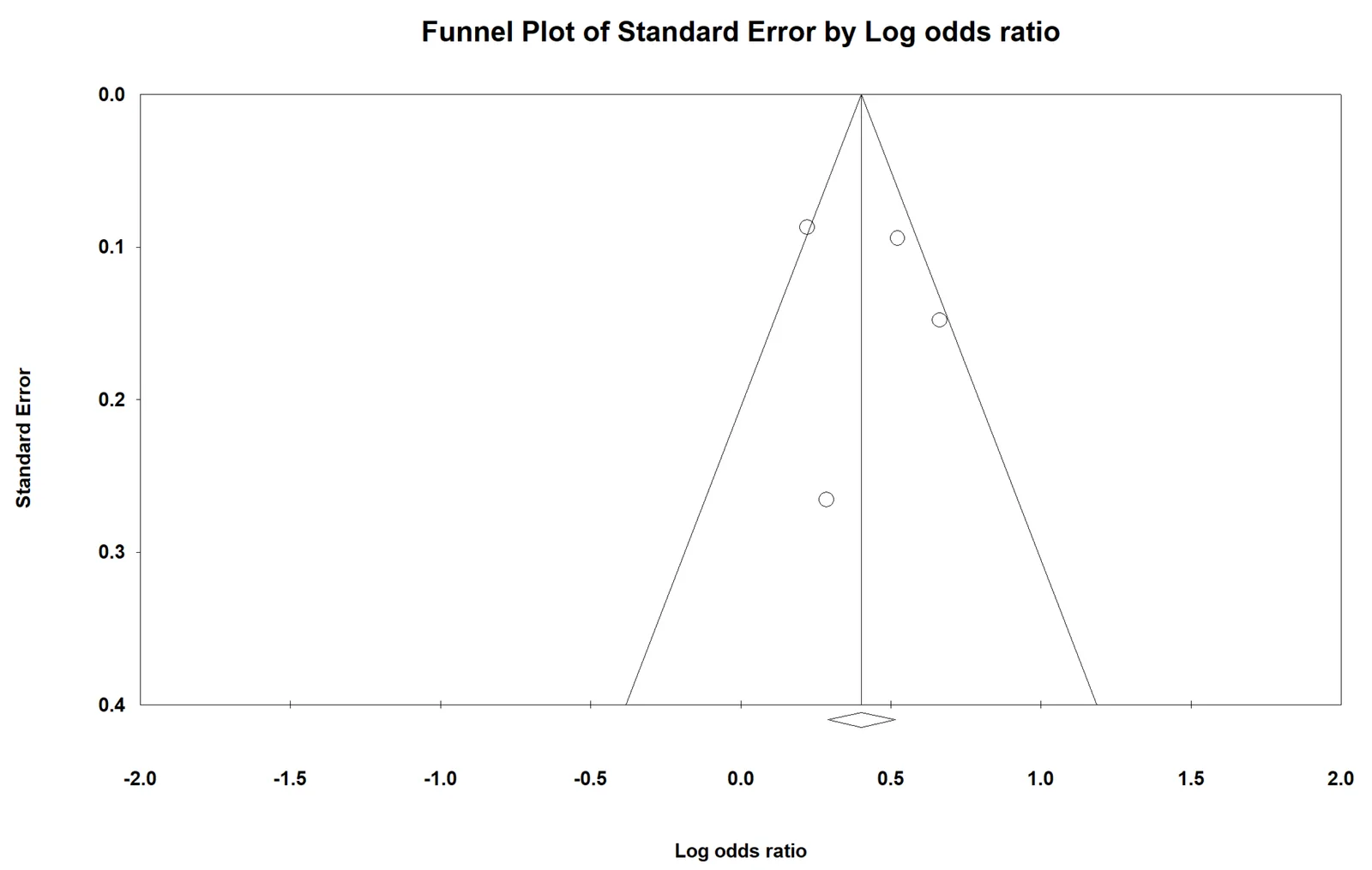
Funnel plot depicting crude ORs for favorable functional outcomes at 3 months after stroke onset.

**Figure 20:**
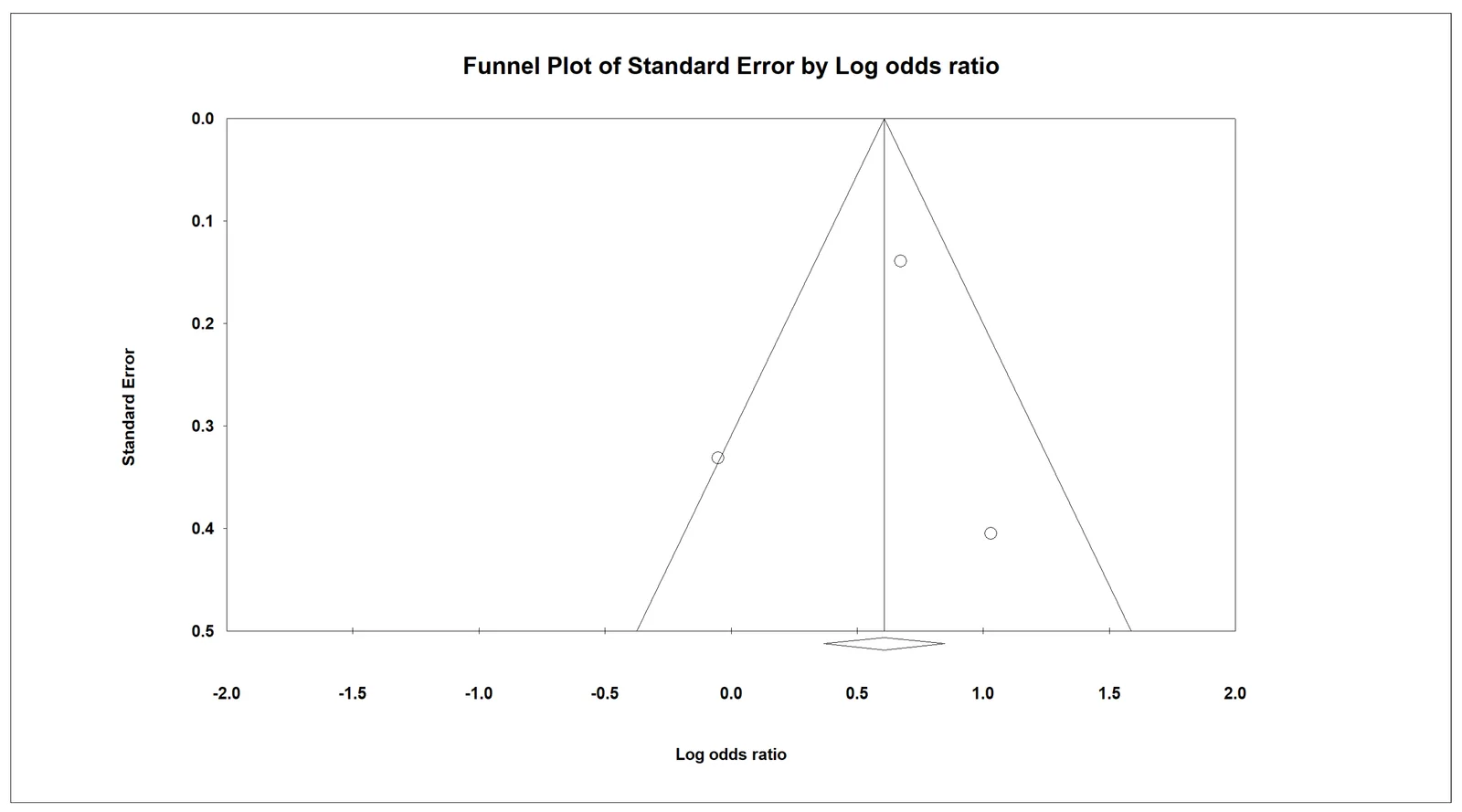
Funnel plot depicting adjusted ORs for favorable functional outcomes at 3 months after stroke onset.

**Table 1:** Full search strategy.

PubMed	“Ischemic stroke” [MeSH terms] AND “Metformin” [All Fields] AND “modified Rankin scale” [All Fields]
Embase	#10 #3 AND #6 AND #9 47
	#9 #7 OR #6 49545
	#8 ‘modified Rankin Scale’/exp 45519
	#7 modified Rankin Scale 36055
	#6 #4 OR #5 114555
	#5 ‘metformin’/exp 109489
	#4 metformin 114555
	#3 #1 OR #2 840777
	#2 ‘stroke’/exp 840777
	#1 ischemic stroke 157887
Web of Science	#4 #1 AND #2 AND #3 12
	#3 TS = (modified Rankin scale) 19468
	#2 TS = (metformin) 50068
	#1 TS = (ischemic stroke) 159412
Cochrane Library	#10 #3 AND #6 AND #9 3
	#9 #7 OR #8 5624
	#8 MeSH descriptor: [modified Rankin Scale] explode all trees 0
	#7 modified Rankin Scale 5624
	#6 #4 OR #5 14786
	#5 MeSH descriptor: [metformin] explode all trees 5609
	#4 metformin 14786
	#3 #1 OR #2 22628
	#2 MeSH descriptor: [ischemic stroke] explode all trees 2272
	#1 ischemic stroke 22628

**Table 2:** Characteristics of the included studies.

Study (First Author, Year of Publication)	Country of Enrollment	Center(s) of Enrollment (Number)	Study Design (Number of Participants)	Reperfusion Therapy	Age of Stroke Onset (Years Old) (Mean, SD)	Gender (Percentage of Male)	HbA1c at Admission (%) (Mean, SD)	Serum Glucose at Admission (mg/dL) (Mean, SD)	Stroke Subtype (Cardio-Embolic) (%)	Serum Creatinine Level (mg/dL) (Mean, SD)
Mima, 2016 [38]	Japan	1	Cohort study (355)	NR	70.1, 10.6	72	7.5, 1.5	178, 70	13.5	NR
Allahverdiyev, 2020 [42]	Turkey	1	Cohort study (70)	NR	69.4, 10.9	48.6	8.4, 2.4	150.7, 72.3	27.1	NR
Westphal, 2020 [40]	Switzerland, Germany, Finland, Italy, Netherlands, Serbia, France, Sweden,	15	Cohort study (1514)	IVT	71.6, 10.2	61.8	NR	169.2, 66.6	39.1	1.01, 0.6
Akhtar, 2022 [32]	Qatar	1	Cohort study (2157)	IVT, EVT, medication	54.4, 13.1	81.2	7.6, 3.5	NR	14.2	NR
Currò, 2022 [34]	Italy	1	Cohort study (170)	IVT, EVT	76.7, 8.7	49.4	NR	185.3, 69.5	34.7	1.1, 0.6
Kersten, 2022 [36]	Netherlands	2	Cohort study (937)	IVT, EVT, medication	75.4, 10.4	53.1	NR	172.8, 54	NR	NR
Tu, 2022 [39]	China	232	Cohort study (7587)	IVT, EVT, medication	65.3, 11.9	57.4	NR	NR	NR	NR
Jian, 2023 [35]	China	1	Cohort study (309)	IVT, EVT, medication	63.3, 11.9	68.3	8.3, 2.1	223.2, 81	6.5	0.7, 0.2
Akiyama, 2024 [33]	Japan	1	Cohort study (160)	IVT, medication	73.7, 8.2	76.2	7.1, 0.9	163.7, 62.1	13.1	0.9, 0.4
Kim, 2024 [37]	Korea	4	Cohort study (231)	EVT	71.7, 12.2	54.1	7.4, 1.5	NR	58.9	1.1, 0.9
Zhang, 2024 [41]	The USA	1	Cohort study (906)	IVT, EVT, medication	69.5, 12.0	56.5	7.6, 1.7	190.9, 60.7	NR	1.5, 1.1

Abbreviations: NR, not reported; IVT, intravenous thrombolysis; EVT, endovascular thrombectomy.

**Table 3:** Certainty of evidence for effect of metformin on favorable functional outcomes at 3 months after stroke.

Quality Assessment						Summary of Findings, Odds Ratio (95% CI)	
Number of Participants (Studies), Follow-Up Period	Risk of Bias	Inconsistency	Indirectness	Imprecision	Publication Bias	Favorable functional outcome (mRS 0–2)	Certainty of Evidence
5322 (4), 3 months	No serious limitation ^a^	Serious limitation ^b^	No serious limitation ^c^	No serious limitation ^d^	Undetectable	1.536 (1.231, 1.915)	Very low ⨁◯◯◯

Abbreviations: mRS, modified Rankin Scale. ^a^ Most studies had a low risk of bias. ^b^ The *I*^2^ value was >50%. ^c^ No indirectness was detected in this outcome. ^d^ Both the upper and lower limits of 95% CI favored metformin use.

**Table 4:** Certainty of evidence for the effect of metformin on neurological severity of stroke at admission.

Quality Assessment						Summary of Findings, SMD (95% CI)	
Number of Participants (Studies), Follow-Up Period	Risk of Bias	Inconsistency	Indirectness	Imprecision	Publication Bias	Neurological severity of stroke (NIHSS score)	Certainty of Evidence
12,934 (8), none	No serious limitation ^a^	Serious limitation ^b^	No serious limitation ^c^	No serious limitation ^d^	Undetectable	−0.138 (−0.230, −0.046)	Very low ⨁◯◯◯

Abbreviations: NIHSS, National Institutes of Health Stroke Scale ^a^ Most studies had a low risk of bias. ^b^ The *I*^2^ value was >50%. ^c^ No indirectness was detected in this outcome. ^d^ Both the upper and lower limits of 95% CI favored metformin use.

**Table 5:** Certainty of evidence for effect of metformin on post-intravenous thrombolysis symptomatic intracranial hemorrhage.

Quality Assessment						Summary of Findings, Odds Ratio (95% CI)	
Number of Participants (Studies), Follow-Up Period	Risk of Bias	Inconsistency	Indirectness	Imprecision	Publication Bias	Symptomatic intracranial hemorrhage	Certainty of Evidence
3841 (3), none	No serious limitation ^a^	No serious limitation ^b^	No serious limitation ^c^	Serious limitation ^d^	Undetectable	0.989 (0.440, 2.221)	Very low ⨁◯◯◯

^a^ Most studies had a low risk of bias. ^b^ The *I*^2^ value was <50%. ^c^ No indirectness was detected in this outcome. ^d^ The upper limit of 95% CI did not favor metformin use, and the lower limit of 95% CI favored metformin use.

**Table 6:** Certainty of evidence for effect of metformin on mortality at 3 months after stroke onset.

Quality Assessment						Summary of Findings, Odds Ratio (95% CI)	
Number of Participants (Studies), Follow-Up Period	Risk of Bias	Inconsistency	Indirectness	Imprecision	Publication Bias	Symptomatic intracranial hemorrhage	Certainty of Evidence
3841 (3), 3 months	No serious limitation ^a^	No serious limitation ^b^	No serious limitation ^c^	No serious limitation ^d^	Undetectable	0.635 (0.511, 0.789)	Low ⨁⨁◯◯

^a^ Most studies had a low risk of bias. ^b^ The *I*^2^ value was <50%. ^c^ No indirectness was detected in this outcome. ^d^ Both the upper and lower limits of 95% CI favored metformin use.

## Data Availability

The original contributions presented in this study are included in the article/Supplementary Materials. Further inquiries can be directed to the corresponding author.

## Supplementary Materials

The following supporting information can be downloaded at: https://www.mdpi.com/article/10.3390/jcm15197532/s1, Table S1: PRISMA Checklist.

## Abbreviations

CKD	Chronic kidney disease
IVT	Intravenous thrombolysis
mRS	Modified Rankin Scale
NIHSS	National Institutes of Health Stroke Scale
OAD	Oral antidiabetic drug (OAD)
sICH	Symptomatic intracranial hemorrhage
T2DM	Type 2 diabetes mellitus
